# The Impact of Steatosis on the Outcome of Liver Transplantation: A Meta-Analysis

**DOI:** 10.1155/2019/3962785

**Published:** 2019-05-14

**Authors:** Qiong-Yue Zhang, Qiong-Fang Zhang, Da-Zhi Zhang

**Affiliations:** Key Laboratory of Molecular Biology for Infectious Diseases (Ministry of Education), Institute for Viral Hepatitis, Department of Infectious Diseases, The Second Affiliated Hospital, Chongqing Medical University, Chongqing, China

## Abstract

**Background and Aims:**

Liver transplantation is one of the most effective treatments for end-stage liver disease as well as for cases of acute liver failure. Facing organ donor shortage, liver transplant teams had to use marginal organs. Thus, increasing availability is a key concern of donor liver grafts including steatotic livers. However, the use of steatotic liver is still controversial. The aim of this systematic review and meta-analysis was to analyze the impact of steatosis on the outcome of liver transplantation.

**Methods:**

We searched PubMed, Cochrane Library, Embase, Web of knowledge, and so on for studies published through May 31, 2018, in which patients experienced liver transplantation using fatty liver. All studies extracted outcome indicators, and we draw conclusions by contrasting outcome indicators in different groups of steatosis. Odds ratios and 95% confidence intervals were calculated. P<0.05 was considered as statistically significant difference.

**Results:**

19 publications were included. There was no significant difference between the group of no steatosis and mild group in primary nonfunction rate (P=0.605) or early graft dysfunction rate (P=0.44). The PNF rate was significantly higher in moderate group (P=0.003) and severe group (P <0.001) compared with that in no steatosis group. The same results were seen in early graft dysfunction rate. However, graft survival rate and patient survival rate did not differ between groups.

**Conclusions:**

Livers with mild steatosis, even with moderate or severe steatosis, could be suitable donor under strict control of transplant conditions.

## 1. Introduction

Liver transplantation (LT) is one of the most effective treatments for end-stage liver disease as well as for cases of acute liver failure. The shortage of donors is a major challenge in liver transplantation, bringing higher waiting-list mortality rate [1]. Therefore, liver transplant teams maximized their search strategies to increase the pool of available liver grafts. Donor pool broadening strategies such as the use of marginal organs, steatosis greater than 30%, donors over 60 years, grafts with a cold ischemia time>12h, donors with hypernatremia, positive serologies for hepatitis B virus (HBV) or hepatitis C virus (HCV), cadaveric split livers, and living donors are being pursed [2].

Currently, the global prevalence of NAFLD is around 25% [3], signifying the pool of potential liver donor is now frequently populated by those with fatty liver disease. However, the use of fatty liver is still controversial. It is believed that donor liver steatosis was associated with a higher risk of primary nonfunction (PNF), early graft dysfunction (EAD), and poor graft survival rate. But, in recent years, several studies demonstrated excellent perioperative and long-term outcomes with the use of grafts with steatosis >30% [4–8]. Therefore, the purpose of this meta-analysis was to analyze the impact of donor steatosis on the outcome of liver transplantation.

## 2. Materials and Methods

This systematic review and meta-analysis was performed based on the Preferred Reporting Items for Systematic Reviews and Meta-analyses (PRISMA) guideline.

### 2.1. Search Strategy

Two researchers independently performed a comprehensive search of PubMed, Embase, the Cochrane library, China National Knowledge Infrastructure, Wanfang database, Google Scholar, “http://cn.Bing.com/academic”, “http://xueshu.baidu.com/”, “http://www.gycc.com/”, Scopus and ISI/web of knowledge database, using the method of free words and subjects. Meanwhile, two researchers independently performed systematic searches in SIGLE, Open Grey, NTlS, and Grey Net International for gray literature to obtain more comprehensive, accurate, and objective research conclusions. Key search terms were “fatty liver”, “steatosis”, “nonalcoholic fatty liver disease donor”, and “liver transplantation”. Databases were searched from the earliest data to 31 May 2018.

### 2.2. Study Selection

We included all liver transplant patients with fatty liver as donor for research. We excluded researches that could not extract valid data, studies where the degree of steatosis was not clear, review articles, case reports, letters, editorials, cohorts with fewer than 5 patients, studies published only as abstracts, nonhuman studies, and non-English language studies. The two researchers searched the literature individually and any disputes were discussed or agreed by the third senior author until the opinions were agreed.

### 2.3. Data Extraction

The two researchers separately extracted literature-related data: (1) the name of the first author, (2) publication year, (3) country, (4) number of patients, (5) study design, (6) the enrolment period, (7) follow-up period, and (8) outcomes.

The outcomes of interest we concerned were as follows: primary nonfunction (PNF) rate, early graft dysfunction (EAD) rate, graft survival rate, patient survival rate. PNF was defined as the need for urgent retransplantation when a graft never demonstrated any evidence of initial function following transplantation in the absence of any vascular complication [9]. EAD was defined as follows: (1) bilirubin 170 mmol/L on postoperative day (POD) 7; (2) international normalized ratio (INR) 1.6 on POD 7; (3) aminotransferase level (alanine aminotransferase [ALT] or aspartate aminotransferase [AST]) >2000 IU/ml from day 1 to day 7 after LT [10].

### 2.4. Quality and Risk of Bias Assessment

The quality of the studies was assessed using a modified Newcastle-Ottawa scale [11]. The Newcastle-Ottawa Quality Assessment Form was used to evaluate the study quality for case-control studies and cohort studies. There are three parts in this assessment form, including selection, comparability, and outcome. Selection, comparability, and outcome domain can be given a maximum of 4, 2, 3 points, respectively. A study scoring a total of 8 or 9 points can be regarded as a high-quality study. A study scoring fewer than 6 points can be regarded as a low-quality study. Other studies can be regarded as moderate-quality studies. We used a fixed-effects model to obtain a summary estimate of outcomes of interest when there is no significant evidence of statistical heterogeneity among the studies. Differences between groups are expressed as odds ratios (ORs) with 95% confidence intervals (CIs). A two-sided P value of <0.05 was considered as statistically significant. Heterogeneity derived from included studies was evaluated using a Chi-squared based I^2^ statistic. The following cut-offs were used to quantify heterogeneity with the I^2^ statistic: 0±25%, low; 25±50%, moderate; and >50%, high heterogeneity [12]. Statistical analysis was performed using STATA 12 statistical software (STATA Corp, College Station, Texas, USA).

## 3. Results

### 3.1. Study Selection

**Figure 1:**
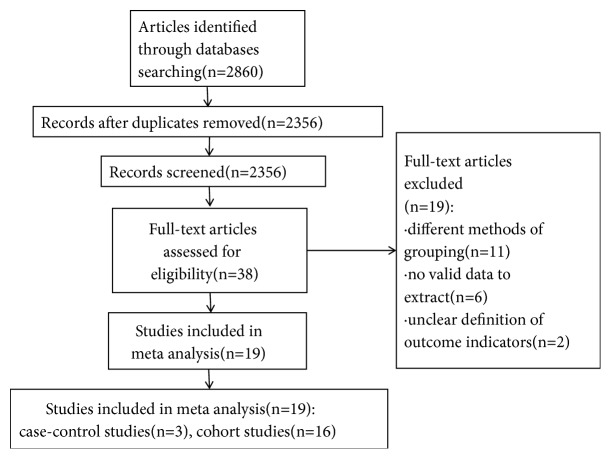
The flow chart of the selection of the studies.

Our initial search identified 2860 references according to the agreement for study selection, no studies gained from Chinese database according to the agreement for study selection, of which 38 full-text articles were reviewed. We excluded 19 studies including different methods of grouping (11 studies), no valid data to extract (6 studies), and unclear definition of outcome indicators (2 studies). Finally, the remaining 19 [4–8, 13–26] references were considered for inclusion. The flow chart of the selection of studies for inclusion in the meta-analysis was shown in Figure 1.

### 3.2. Study Characteristics

We included 19 studies in the present meta-analysis according to the inclusion and exclusion criteria, including 3 case-control studies and 16 cohort studies. A total of 4002 patients enrolled. The main characteristics of the studies were shown in Table 1.

### 3.3. Quality Assessment and Risk of Bias

The quality of the studies was shown in Supplementary Table 1 in details. 6 studies scoring 8 or 9 are considered high quality. Among these, 3 case-control studies were all regarded as high quality. 4 studies scoring fewer than 6 are considered low quality. Other studies were regarded as moderate-quality studies.

### 3.4. Outcomes Analyses

The experiments were grouped according to the degree of steatosis, including no steatosis, mild (<30%) steatosis, moderate (30-60%) steatosis, and severe (>60%) steatosis [26]. For the convenience of expression, we defined the group of no steatosis as control group. At the same time, we defined the group of mild steatosis, the group of moderate steatosis, and the group of severe steatosis as mild group, moderate group, and severe group. The outcomes of liver transplantation were evaluated by the following parameters: PNF rate, EAD rate, graft survival rate, and patient survival rate. We determined the impact of steatosis on posttransplant outcomes by comparing differences in outcomes between groups.

#### 3.4.1. Primary Nonfunction Rate

**Figure 2:**
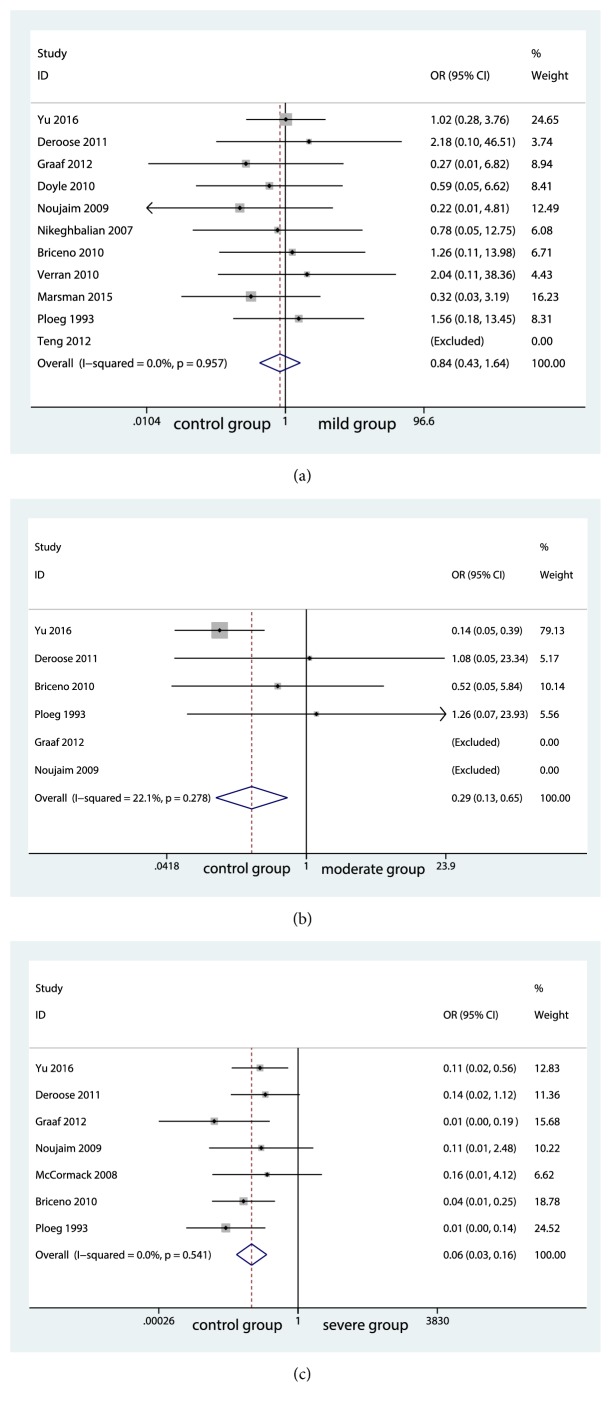
Forest plot for primary nonfunction rates among groups.

10 studies reported PNF rate. PNF rate was found to be comparable between the group of no steatosis (control group) and the group of mild steatosis (mild group) (OR=0.52; 95% CI, 0.429 to 1.639; P=0.605; Figure 2(a)). No significant risk of publication bias was found as shown in Supplementary Figure 1. However, significant differences were seen between the group of no steatosis (control group) and the group of moderate steatosis (moderate group) (OR=2.99; 95% CI, 0.128 to 0.652; P=0.003; Figure 2(b)). Similar result was also found between control group and the group of severe steatosis (severe group) (OR=6.03; 95% CI, 0.026 to 0.156; P<0.001; Figure 2(c)). No significant heterogeneity among the studies was found and the fixed-effects model was performed shown in Figure 2.

#### 3.4.2. Early Graft Dysfunction Rate

**Figure 3:**
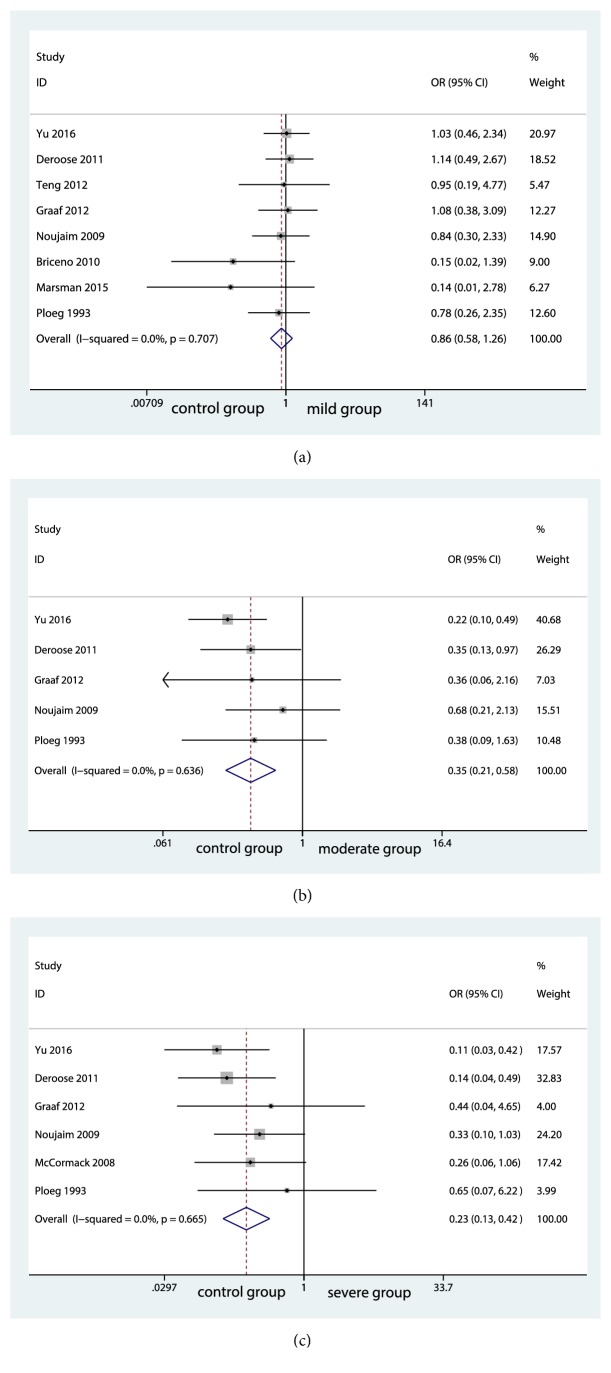
Forest plot for early graft dysfunction rates among groups.

The meta-analysis including 8 studies showed no significance in EAD rate between control group and mild group (OR=0.77; 95% CI, 0.583 to 1.265; P=0.44; Figure 3(a)). Without significant evidence of statistical heterogeneity among the studies, the fixed-effects model was applied (I^2^=0.0%; P=0.707; Figure 3(a)). No significant risk of publication bias was found and details were shown in Supplementary Figure 2. Similar to the outcome of PNF rate, the EAD rate was significantly higher in moderate group compared with control group (OR=4.07; 95% CI, 0.215 to 0.584; P<0.001; Figure 3(b)). The I^2^ statistic (I^2^=0.0%; P=0.636; Figure 3(b)) showed no significant heterogeneity among the studies and the fixed-effects model was applied. Meanwhile, significant differences were seen between control group and severe group (OR=4.84; 95% CI, 0.13 to 0.422; P<0.001; Figure 3(c)). The importance of heterogeneity was minimal (I^2^=0.0%; P=0.665). Thus, fixed-effects model was applied (Figure 3(c)).

#### 3.4.3. Graft Survival Rate

**Figure 4:**
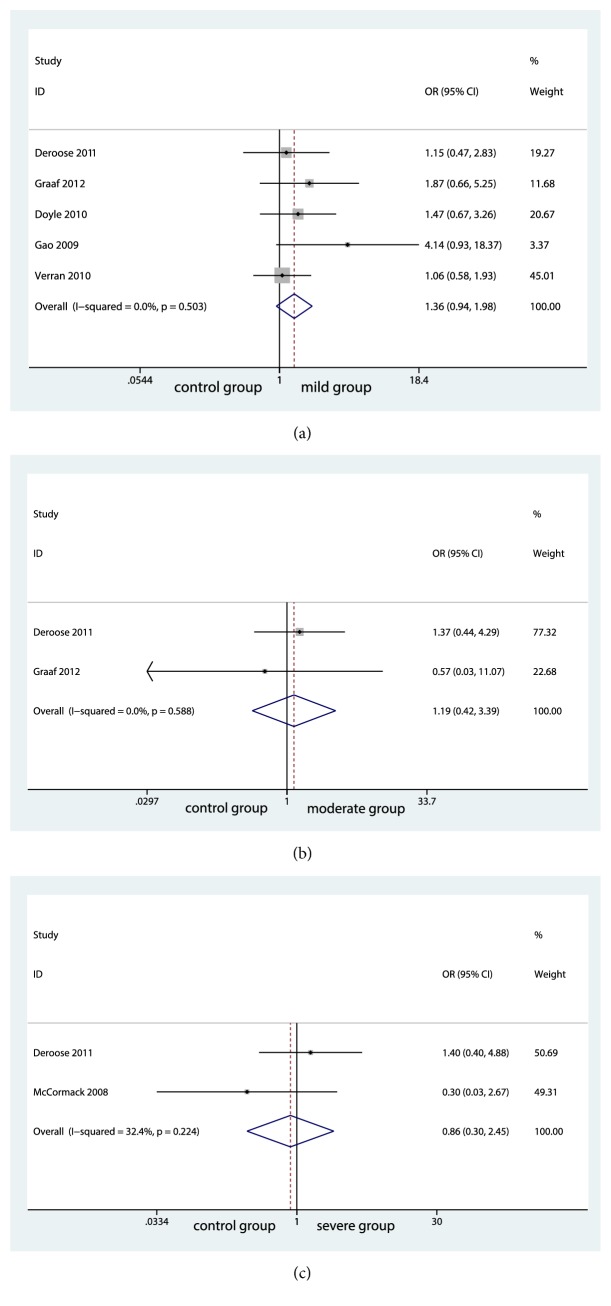
Forest plot for graft survival rates among groups.

We also conducted an analysis for 1-year graft survival rate between groups. Surprisingly, no significantly differences were seen among groups (control group and mild group, OR=1.61; 95% CI, 0.936 to 1.978; P=0.107; Figure 4(a)) (control group and moderate group, OR=0.33; 95% CI, 0.418 to 3.393; P=0.744; Figure 4(b)) (control group and severe group, OR=0.29; 95% CI, 0.298 to 2.454; P=0.771; Figure 4(c)). No significant evidence of statistical heterogeneity was found between control group and mild group (I^2^=0.0%; P=0.503; Figure 4(a)). The same as the heterogeneity between control group and moderate group (I^2^=0.0%; P=0.588; Figure 4(b)). The fixed-effects model was applied. No significant heterogeneity was found among the studies and fixed-effects model was used (I^2^=32.4%; P=0.224; Figure 4(c)). Further meta-analysis was limited by small sample size, although we extracted the graft survival rate of one month, three months, six months, and 1 year.

#### 3.4.4. Patient Survival Rate

**Figure 5:**
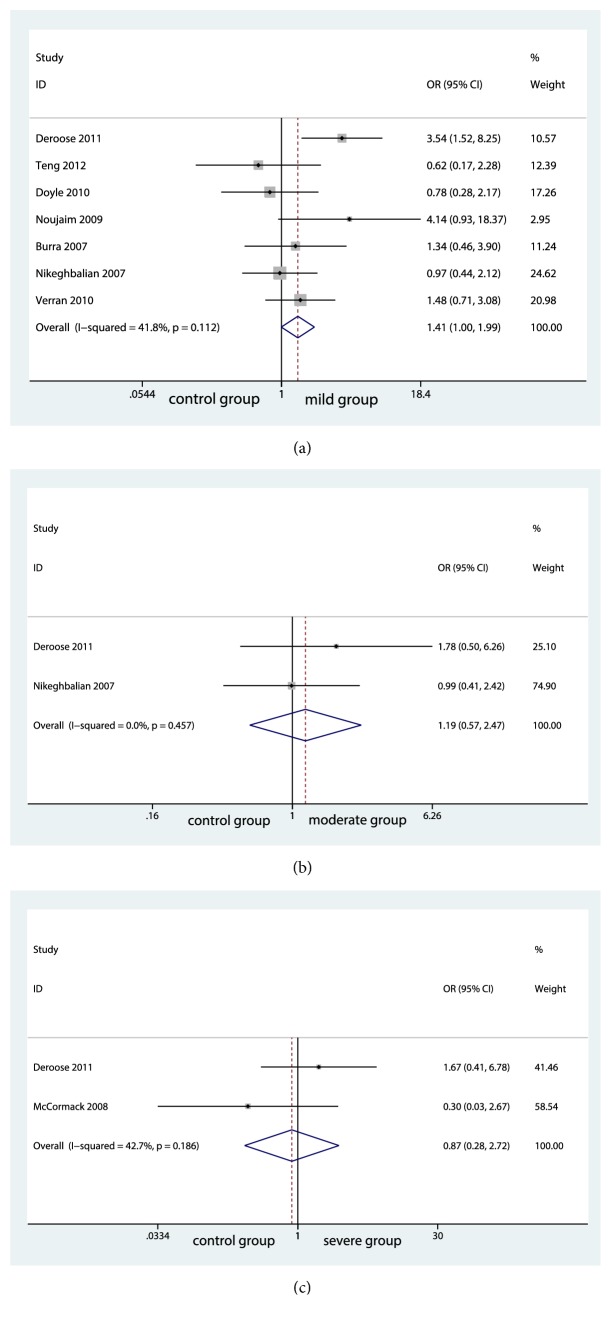
Forest plot for 1-year patient survival rates among groups.

**Figure 6:**
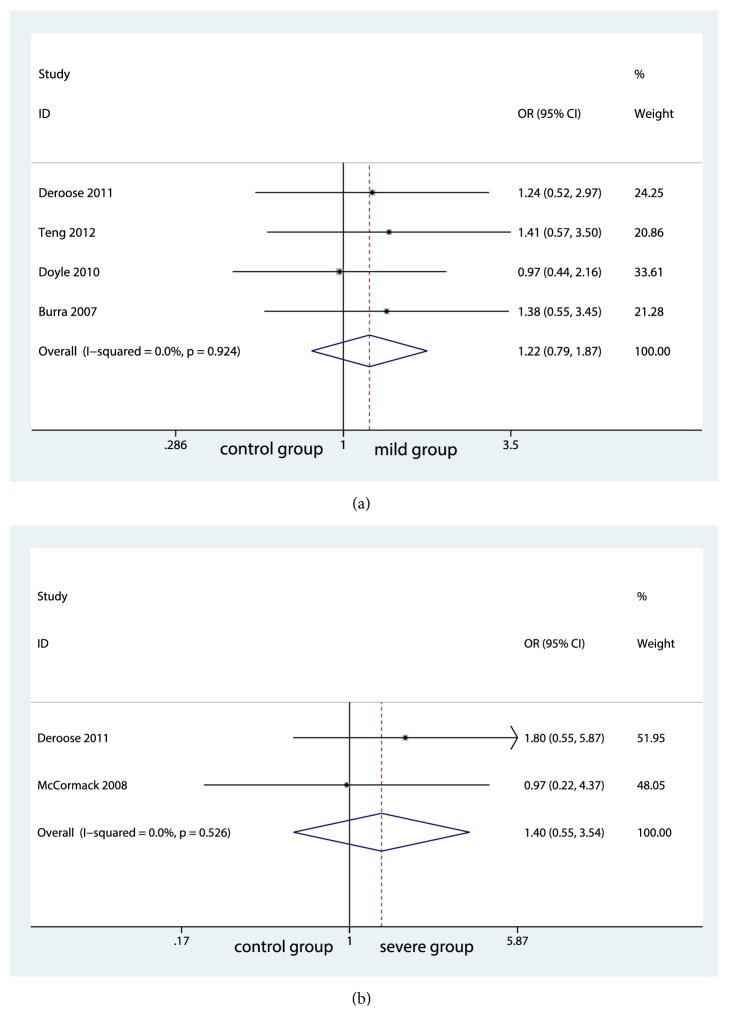
Forest plot for 3-year patient survival rates among groups.

We extracted data of 1-year patient survival rate, 3-year patient survival rate, 5-year patient survival rate, and 10-year patient survival rate. Ultimately, 1-year and 3-year patient survival rates were analyzed in the present meta-analysis. No significant difference was found among groups in 1-year survival rate (control group and mild group, OR=1.94; 95% CI, 0.996 to 1.992; P=0.053; Figure 5(a)) (control group and moderate group, OR=0.46; 95% CI, 0.572 to 2.465; P=0.644; Figure 5(b)) (control group and severe group, OR=0.25; 95% CI, 0.275 to 2.721; P=0.805; Figure 5(c)). There was a low statistical heterogeneity among the studies and fixed-effects model was applied. For 3-year patient survival, the outcome was analyzed in four studies with a low heterogeneity between control group and mild group (I^2^=0.0%, P=0.924, Figure 6(a)), which did not differ significantly between them (OR=0.89; 95% CI, 0.789 to 1.873; P=0.376; Figure 6(a)). Also, there was no significant differences between control group and severe group (OR=0.71; 95% CI, 0.554 to 3.545; P=0.476; Figure 6(b)) with a minimal heterogeneity (I^2^=0.0%; P=0.526; Figure 6(b)). However, further analysis between control group and moderate group was unable to be analyzed because of insufficient size of studies.

## 4. Discussion

Liver transplantation (LT) is an established life-saving operation for patients with end-stage liver disease and acute liver failure. Imbalance between organ shortage and higher waiting-list mortality rate persuaded liver transplant team to use marginal donor liver to expand the donor pool. The aim of this meta-analysis was to examine the impact of steatosis on the outcome of liver transplantation.

As we know, there are two patterns of hepatic steatosis, microvascular and macrovesicular. Microvascular steatosis refers to the accumulation of tiny lipid droplets measuring <1 mm giving a foamy appearance of the cytoplasm. It is commonly considered that microsteatosis is not relevant when selecting liver grafts for LT. Macrovesicular steatosis is defined by the presence of small to large droplets that may end up occupying the whole cytoplasm. The volume of large droplet macrosteatosis in the liver graft is closely linked to its suitability for transplantation. There were a large number of studies about macrovesicular steatosis, accounting for a large proportion of our research. We included an article about microvascular in the analysis. Since no unacceptable heterogeneity was observed and there was no significant difference for the results after removing the article, we reserved it.

EASL Clinical Practice Guidelines [27] proposed that mild macrosteatosis (<30% volume) was considered suitable for transplantation. This statement was consistent with the results of our analysis. We did not observe higher primary nonfunction rates and early graft dysfunction rates in mild group compared with control group. Simultaneously, liver with mild steatosis did not increase patient survival rates and grafts survival rates. These findings supported that liver with mild steatosis can be used as a safe donor for liver transplantation.

For liver with moderate steatosis, large registry studies revealed that moderate steatosis was an independent prognostic factor for poor postoperative outcomes [13, 14] whereas other authors still suggested that the use of livers with moderate steatosis in well-controlled cases can be successful [5, 17]. Our analysis showed that moderate steatosis had no significant effect on survival rates compared with control group in spite of poor graft function. Our results provided a proof that livers with moderate steatosis can also be suitable liver grafts.

Severely steatotic liver grafts were all discarded in 1990s. Spitzer et al. [28] suggested that macrovesicular steatosis >30% was an independent risk factor in the Donor Risk Index (DRI) lists. In recent years, several studies demonstrated that livers with >60% macrovesicular steatosis were still acceptable for LT [8, 29]. Wong et al. [29] had observed excellent outcomes of using grafts with macrovesicular steatosis >60% from brain-dead donors. The early postoperative outcomes of using severely steatotic liver grafts were impeccable with no hospital mortality, no PNF, and no EAD. Chavin et al. [8] carried out a research about long-term outcomes affected by the degree of donor steatosis. The overall patient survival rates and graft survival rates were similar among groups across all time points including 30-day, 1-yr, 3-yr, 5-yr, and 10-yr survival. In our study, similar to the posttransplantation outcome of moderate group, we found a significantly higher PNF rate and EAD rate in severe group while no significant difference was seen in survival rates between severe group and control group. Thus, liver with severe steatosis could also be considered as liver grafts.

A possible explanation for the different outcomes could be advanced donor age, graft donation after cardiac death, prolonged donor warm ischaemia time (DWIT), and prolonged cold ischemia time (CIT). And CIT was considered as a key factor among them. It is well known that hepatic steatosis is defined as the accumulation of droplets of fat in the hepatocytes. The narrowed sinusoidal lumens caused by swollen fatty hepatocytes increase intrahepatic vascular resistance and, thus, decrease the sinusoidal flow which contributes to the persistent state of chronic cellular hypoxia predisposing the steatotic liver to ischemia/reperfusion injury (I/R injury). This ischemia/reperfusion injury initiates a sequence of events leading to cellular damage with early graft dysfunction. However, Westerkamp et al. [5] observed favorable postoperative outcomes of moderate steatotic livers by minimizing the effects of I/R injury using a strict policy to keep the CIT as short as possible. Similarly, Wong et al. [29] found that with a CIT>7 hours, the peak AST in severely steatotic grafts was much higher than that in the controls and that in severely steatotic livers with a CIT<7 hours. In addition, Tekin et al. [30] considered that marginal livers should be transplanted within 12 hours attributable to increased CIT, leading high risk of developing early graft dysfunction. These findings suggested that the use of steatotic livers even with moderate and severe steatosis following a strict protocol to keep the CIT as short as possible may expand the donor pool.

Apart from the preservation methods used to protect the organ from IR injury, the final posttransplantation outcome could also be linked to the changes of hepatocyte. Interestingly, several studies reported reversal or disappear of steatosis. Westerkamp et al. [5] found the reversal of steatosis in 82% of the donor livers with a follow-up biopsy. The degree of fatty infiltration decreased from 60-30% to 10% or less. Another study [23] demonstrated complete dissipation of steatosis in the steatosis>35% group and 85% of the moderately steatotic grafts when biopsies were performed more than 10 days after liver transplantation. Similar results were also observed in animal experiments although the mechanism has not yet to be fully elucidated. These findings revealed that steatosis was not a permanent injury to the liver parenchyma. Patients can achieve good survival rates after recovering from the acute insult. Hence, moderate-to-severe steatotic livers still have the potential to be safely utilized instead of being discarded.

Our meta-analysis must be considered in light of several important limitations. Firstly, a degree of selection bias has been generated as we excluded studies published in other languages or databases. Despite this, with only a small number of studies focusing on the posttransplant outcomes of moderate-to-severe steatosis, a thoroughly meta-analysis evaluating the long-term survival rates affected by the degree of donor steatosis was unable to be performed. Thus, further high-quality studies will still be needed to confirm these results.

In conclusion, livers with mild steatosis were safe liver grafts. Moderately and severely steatotic livers could also produce favorable postoperative outcomes with a strict protocol to keep the CIT as short as possible without decreasing graft survival rate and patient survival rate. Our meta-analysis to some degree expanded the use of livers with steatosis in liver transplantation. This may further provide a potential solution for the shortage of liver donor in the future.

## Figures and Tables

**Table 1:** The main characteristics of the included studies.

Author	Year	Country of origin	Number of transplants	Type of study†	Enrolment period	follow-up period
Andert et al [4]	2017	Germany	94	Co, P	2010-2016	August 2016

Westerkamp et al [5]	2015	The Netherlands	126	Co, P	2000-2012	July 2013

Yu et al [6]	2017	China	563	Co, P	2010-2014	August 2010

Deroose et al [7]	2011	Netherlands	185	Co, P	2000-2004	September 2015

Chavin et al [8]	2013	USA	116	Co, P	1999-2001	at least 10-year or graft loss or death

Teng et al [13]	2012	China	131	Co, Re	2007-2008	February 2011

Graaf et al [14]	2012	Australia	255	Co, Re	2001-2007	ns

Doyle et al [15]	2010	USA	310	Co, P	2002-2008	5 years

Noujaim et al [16]	2009	Brazil	118	Co, P	2002-2008	21±19.5 months

Gao et al [17]	2009	China	48	CaCo, Re	2003-2005	1 year

Frongillo et al [18]	2009	Italy	24	Co, Re	2000-?	patient death or re-transplantation

Burra et al [19]	2009	Italy	116	Co, P	1999-2001	3 years

Nikeghbalian et al [20]	2007	Iran	174	Co, Re	1993-2006	1 year

McCormack et al [21]	2007	Switzerland	60	CaCo, Re	2002-2006	3 years

Perez-Daga et al [22]	2006	Spain	300	Co, Re	1997-2004	20000+days

Briceno et al [23]	2005	Spain	500	Co, Re	ns‡	90 days

Verran et al [24]	2003	Australia	443	Co, Re	1986-2000	14 months

Marsman et al [25]	1996	USA	116	CaCo, Re	1990-1994	1167 days

Ploeg et al [26]	1993	USA	323	Co, Re	1984-1991	ns

Type of study† (Co: cohort study, CaCo: case-control study, CaR: case report, P: prospective, Re: retrospective)

ns‡: not stat

## Supplementary Materials

Supplementary Materials Supplemental method Supplementary Table 1. The quality of the studies included. Supplemental result Supplementary Figure 1. Comparison-adjusted funnel plot for the primary nonfunction rate between control group and mild group. Supplementary Figure 2. Comparison-adjusted funnel plot for the early graft dysfunction rate between control group and mild group.

## References

[1] Fisher R. A. (2017). Living donor liver transplantation: eliminating the wait for death in end-stage liver disease?. *Nature Reviews Gastroenterology & Hepatology*.

[2] Jiménez-Romero C., Caso Maestro O., Cambra Molero F. (2014). Using old liver grafts for liver transplantation: where are the limits?. *World Journal of Gastroenterology*.

[3] Ng M., Fleming T., Robinson M., et al (2014). Global, regional, and national prevalence of overweight and obesity in children and adults during 1980–2013: a systematic analysis for the Global Burden of Disease Study 2013. *The Lancet*.

[4] Andert A., Ulmer T. F., Schoning W. (2017). Grade of donor liver microvesicular steatosis does not affect the postoperative outcome after liver transplantation. *Hepatobiliary & Pancreatic Diseases International*.

[5] Westerkamp A. C., De Boer M. T., Van Den Berg A. P., Gouw A. S. H., Porte R. J. (2015). Similar outcome after transplantation of moderate macrovesicular steatotic and nonsteatotic livers when the cold ischemia time is kept very short. *Transplant International*.

[6] Yu Z., Yu S., Zhang L. (2017). Retraction: safety limitations of fatty liver transplantation can be extended to 40%: experience of a single centre in China. *Liver International: Official Journal of the International Association for the Study of the Liver*.

[7] Deroose J. P., Kazemier G., Zondervan P., IJzermans J. N. M., Metselaar H. J., Alwayn I. P. J. (2011). Hepatic steatosis is not always a contraindication for cadaveric liver transplantation. *HPB*.

[8] Chavin K. D., Taber D. J., Norcross M. (2013). Safe use of highly steatotic livers by utilizing a donor/recipient clinical algorithm. *Clinical Transplantation*.

[9] Johnson S. R., Alexopoulos S., Curry M., Hanto D. W. (2007). Primary nonfunction (PNF) in the MELD era: An SRTR database analysis. *American Journal of Transplantation*.

[10] Olthoff K. M., Kulik L., Samstein B. (2010). Validation of a current definition of early allograft dysfunction in liver transplant recipients and analysis of risk factors. *Liver Transplantation*.

[11] Stang A. (2010). Critical evaluation of the Newcastle-Ottawa scale for the assessment of the quality of nonrandomized studies in meta-analyses. *European Journal of Epidemiology*.

[12] Higgins J. P. T., Thompson S. G., Deeks J. J., Altman D. G. (2003). Measuring inconsistency in meta-analyses. *British Medical Journal*.

[13] Teng da H., Zhu Z. J., Zheng H. (2012). Effect of steatosis donor liver transplantation on hepatocellular carcinoma recurrence: experience at a single institution. *Hepato-Gastroenterology*.

[14] de Graaf E. L., Kench J., Dilworth P. (2012). Grade of deceased donor liver macrovesicular steatosis impacts graft and recipient outcomes more than the Donor Risk Index. *Journal of Gastroenterology and Hepatology*.

[15] Doyle M. B. M., Vachharajani N., Wellen J. R. (2010). Short- and long-term outcomes after steatotic liver transplantation. *JAMA Surgery*.

[16] Noujaim H. M., de Ville de Goyet J., Montero E. F. (2009). Expanding postmortem donor pool using steatotic liver grafts: a new look. *Transplantation*.

[17] Gao F., Xu X., Ling Q. (2009). Efficacy and safety of moderately steatotic donor liver in transplantation. *Hepatobiliary & Pancreatic Diseases International*.

[18] Frongillo F., Avolio A. W., Nure E. (2009). Quantification of degree of steatosis in extended criteria donor grafts with standardized histologic techniques: implications for graft survival. *Transplantation Proceedings*.

[19] Burra P., Loreno M., Russo F. P. (2009). Donor livers with steatosis are safe to use in hepatitis C virus-positive recipients. *Liver Transplantation*.

[20] Nikeghbalian S., Nejatollahi S. M. R., Salahi H. (2007). Does donor's fatty liver change impact on early mortality and outcome of liver transplantation. *Transplantation Proceedings*.

[21] McCormack L., Petrowsky H., Jochum W., Mullhaupt B., Weber M., Clavien P.-A. (2007). Use of severely steatotic grafts in liver transplantation: a matched case-control study. *Annals of Surgery*.

[22] Perez-Daga J. A., Santoyo J., Suárez M. A. (2006). Influence of degree of hepatic steatosis on graft function and postoperative complications of liver transplantation. *Transplantation Proceedings*.

[23] Cheng Y., Chen C., Lai C. (2001). Assessment of donor fatty livers for liver transplantation. *Transplantation*.

[24] Verran D. J., Kusyk T., Painter D. (2003). Clinical experience gained from the use of 120 steatotic donor livers for orthotopic liver transplantation. *Liver Transplantation*.

[25] Marsman W. A., Wiesner R. H., Rodriguez L. (1996). Use of fatty donor liver is associated with diminished early patient and graft survival. *Transplantation*.

[26] Ploeg R. J., D'Alessandro A. M., Knechtle S. J. (1993). Risk factors for primary dysfunction after liver transplantation—a multivariate analysis. *Transplantation*.

[27] European Association for the Study of the Liver (2016). EASL clinical practice guidelines: liver transplantation. *Journal of Hepatology*.

[28] Spitzer A. L., Lao O. B., Dick A. A. S. (2010). The biopsied donor liver: incorporating macrosteatosis into high-risk donor assessment. *Liver Transplantation*.

[29] Wong T. C. L., Fung J. Y. Y., Chok K. S. H. (2016). Excellent outcomes of liver transplantation using severely steatotic grafts from brain-dead donors. *Liver Transplantation*.

[30] Tekin K., Imber C. J., Atli M. (2004). A simple scoring system to evaluate the effects of cold ischemia on marginal liver donors. *Transplantation*.

