# Supplementary material for: The Impact of Steatosis on the Outcome of Liver Transplantation: A Meta-Analysis
Source: Biomed Res Int. 2019 May 14;2019:3962785. doi: 10.1155/2019/3962785 (PMC6536983; doi:10.1155/2019/3962785)

Contents

Supplemental method (**page 2**)
Supplementary Table 1. The quality of the studies included (page 2).
Supplemental result (page 3)
Supplementary Figure 1. Comparison-adjusted funnel plot for the primary non-function rate between control group and mild group (page 3).
Supplementary Figure 2. Comparison-adjusted funnel plot for the early graft dysfunction rate between control group and mild group (page 3).

Supplementary Table 1.The quality of the studies included.

Case control studies									
	Selection				comparability	Exposure			Total
	1	2	3	4	/	1	2	3	
Gao et al[17]	√	√	√	√	√	√	√	√	8
McCormack et al[21]	√	√	√	√	√	√	√	√	8
Marsman et al[25]	√	√	√	√	√	√	√	√	8

Cohort studies									
	Selection				comparability	Exposure			Total
	1	2	3	4	/	1	2	3	
Andert et al[4]	√	√	√	√	√	√	√		7
Westerkamp et al[5]	√	√	√	√	√	√	√	√	8
Yu et al[6]	√	√	√	√	√	√		√	7
Deroose et al[7]			√	√	√	√	√	√	6
Chavin et al[8]	√	√	√	√	√	√			6
Teng et al[13]	√	√	√	√	√		√	√	7
Graaf et al[14]	√	√	√		√	√	√	√	7
Doyle et al[15]	√	√	√	√	√	√	√	√	8
Noujaim et al[16]		√	√	√	√		√	√	6
Frongillo et al[18]			√		√	√	√	√	5
Burra et al[19]	√	√	√	√	√√	√	√	√	9
Nikeghbalian et al[20]	√	√	√		√		√	√	6
Perez-Daga et al[22]	√	√	√		√				4
Briceno et al[23]				√	√			√	2
Verran et al[24]	√	√	√		√√			√	6
Ploeg et al[26]	√	√	√		√			√	5

Supplementary Figure 1. Comparison-adjusted funnel plot for the primary non-function rate between control group and mild group.

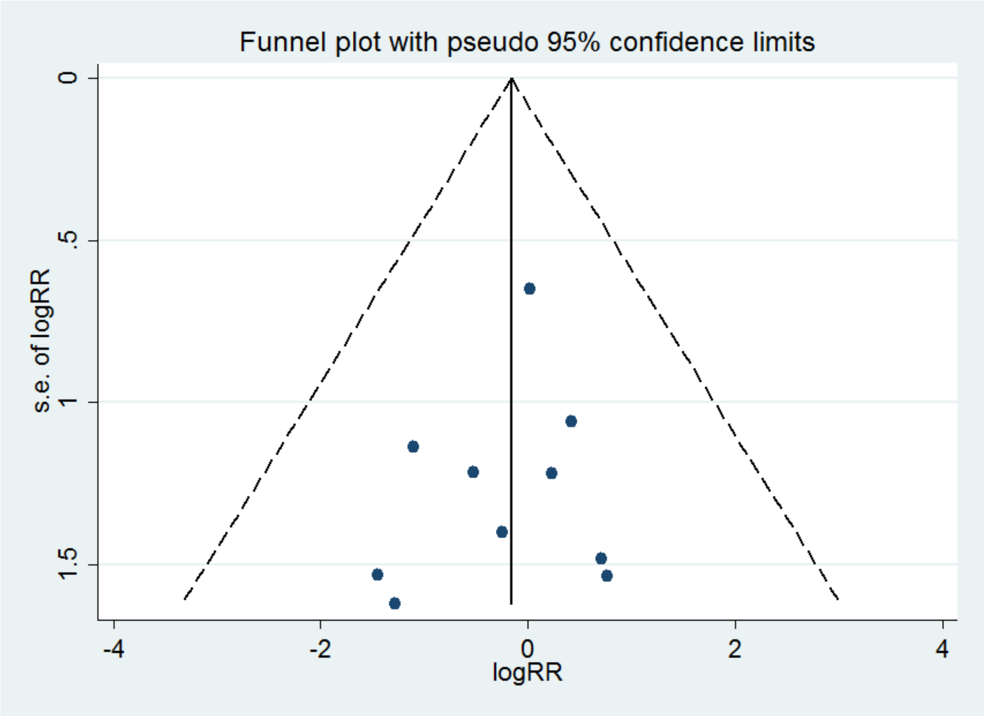

Supplementary Figure 2. Comparison-adjusted funnel plot for the early graft dysfunction rate between control group and mild group.

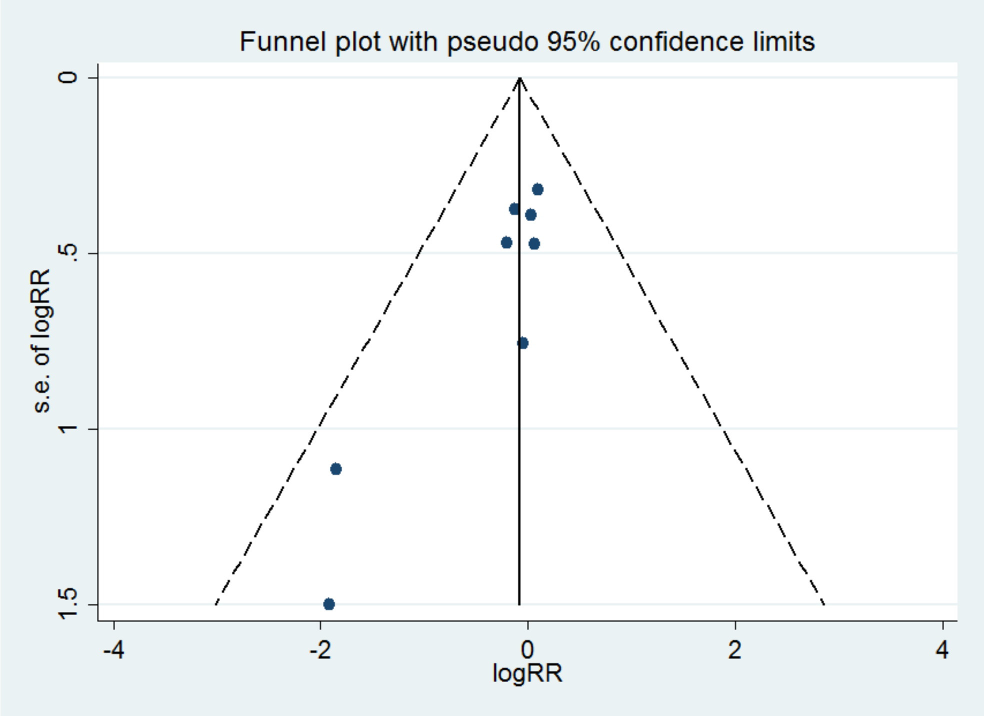

Supplement: Supplementary Materials — Supplemental method Supplementary Table 1. The quality of the studies included. Supplemental result Supplementary Figure 1. Comparison-adjusted funnel plot for the primary nonfunction rate between control group and mild group. Supplementary Figure 2. Comparison-adjusted funnel plot for the early graft dysfunction rate between control group and mild group. [file 3962785.f1.pdf]
